# The arc-technique using a photodynamic nail in a dysmorphic fragility fracture of the posterior pelvic ring – a technical note & case report

**DOI:** 10.1016/j.tcr.2026.101330

**Published:** 2026-04-22

**Authors:** Tobias Fritz, Emmanouil Liodakis, Jonas Gras, Marcel Orth, Antonius Pizanis, Marcus Örgel, Tim Pohlemann, David Osche

**Affiliations:** a Saarland University Medical Center, Kirrbergerstr. 100, 66421, Homburg, Germany

**Keywords:** Fragility fractures, Photodynamic nail, Sacral dysmorphism, Pelvic stabilization, Sacral fractures

## Abstract

**Abstract case:**

This case report presents a novel stabilization approach for managing fragility fractures in the dysmorphic sacrum of a 71-year-old male patient. The technique addresses anatomical complexities associated with sacral dysmorphism by employing a photodynamic intramedullary implant.

**Conclusion:**

The arc-technique utilizing a flexible photodynamic nail enables effective stabilization of the posterior pelvic ring, even in cases involving sacral dysmorphism.

## Introduction

The incidence of fragility and pathological fractures of the pelvis is rising, with projections estimating a 2.4-fold increase by 2030 [1], [2]. While many cases are managed conservatively, surgical stabilization is necessary when non-operative approaches fail. Iliosacral screw fixation is considered the gold standard but poor bone quality and anatomical variability have a severe impact on the surgical results [3], [4].

Alternative implants and techniques such as sacral bars, spinopelvic fixators, the SacroNail and cement-augmented screws have been described, but require a transiliosacral corridor [5], [6], [7], [8]. Pelvises with sacral dysmorphism, present in 25–40% of patients, particularly affects S1 and may preclude standard iliosacral fixation [9], [10], [11]. Therefore, the stabilization of the S1 vertebra is usually achieved by two ascending S1 screws or with special implant designs, like the CurvaFix implant, which was introduced in the US to address these anatomical challenging corridors or using the Silony Verticale system which allows unilateral angular stable fixation.

This case report describes a novel technical approach, using the first application of a transiliosacral photodynamic implant in a dysmorphic sacrum. The arc configuration of the implant allows to create a transiliosacral stabilization and mimics continuous load transmission principles, as used in engineering designs [12], [13], [14].

The patient, a 71-year-old male with metastatic prostate cancer, sustained a pathological pelvic ring fracture. CT imaging revealed a dysmorphic S1 vertebra and metastatic osteolysis in S2, excluding standard screw placement. After a failed conservative approach, the novel arc-technique using photodynamic nails was employed.

## Planning & surgical technique

### Preoperative planning

**Fig. 1:**
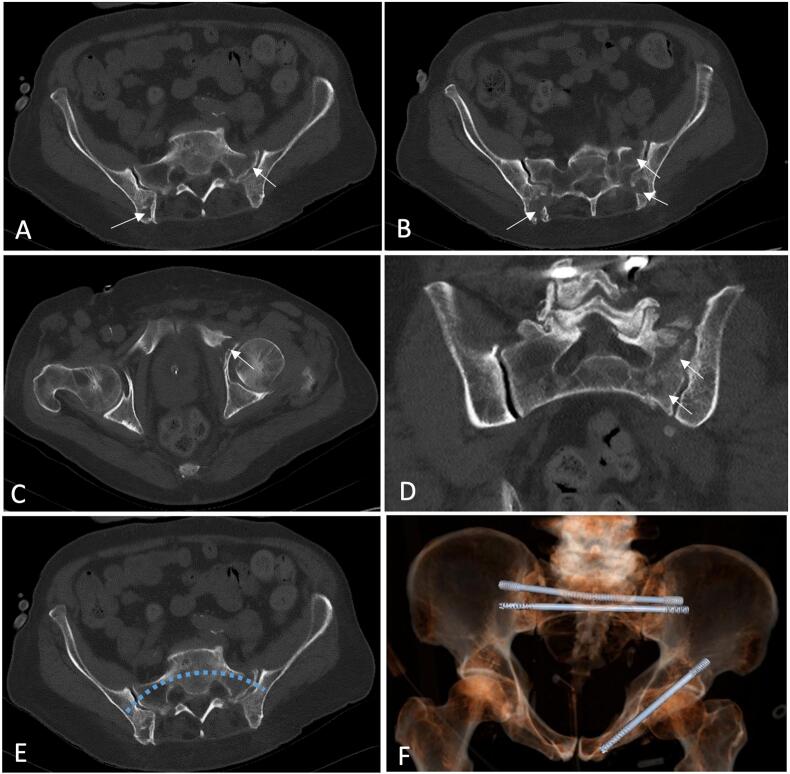
A-D show representative axial CT-scan reconstructions with the fracture lines and bone stock loss marked by the arrows. E shows an axial view with a the planned implant corridor in the S1 body, F shows a 3D-reconstruction with preoperative implant planning (3D-Trauma, Sectra, Sweden). Screw templates were used to mimic the corridors for the photodynamic nail implants, therefore two oblique 8.0 mm corridors were planned to mimic the arc-like configuration of the implant Additionally a transiliosacral corridor was planned in S2 and a antegrade corridor for the left superior pubic ramus was planned.

For preoperative planning, a CT-based 3D imaging with virtual modeling was used (3D-Trauma, Sectra, Sweden). Due to the absence of a curved implant template, oblique screw vectors were simulated to approximate the arc-construct. Lengths were measured as 160 mm for posterior and S2 nails and 120 mm for the pubic ramus (Fig. 1).

### Surgical technique

Under general anesthesia, the patient was positioned prone on a radiolucent carbon table. Intraoperative 3D navigation (Brainlab, Munich, Germany) was employed to optimize implant placement. The dynamic reference base (DRB) was secured to the left posterior iliac crest to avoid interference during subsequent steps.

An intraoperative 3D CT scan (Cios-Spin, Siemens Healthineers, Erlangen, Germany) enabled real-time navigation-assisted planning.

### S1 corridor: arc technique

For the dysmorphic S1 vertebra the novel arc technique was used for the first time. Therefore, the entry point was determined using the optical 3D navigation guide. The entry point for an oblique corridor to S1 can be found at the height of S2, which allows to use the same skin incision on the left side for both the S1 and S2 corridor. The prone position allows a movement without interference with the operation table.

**Fig. 2:**
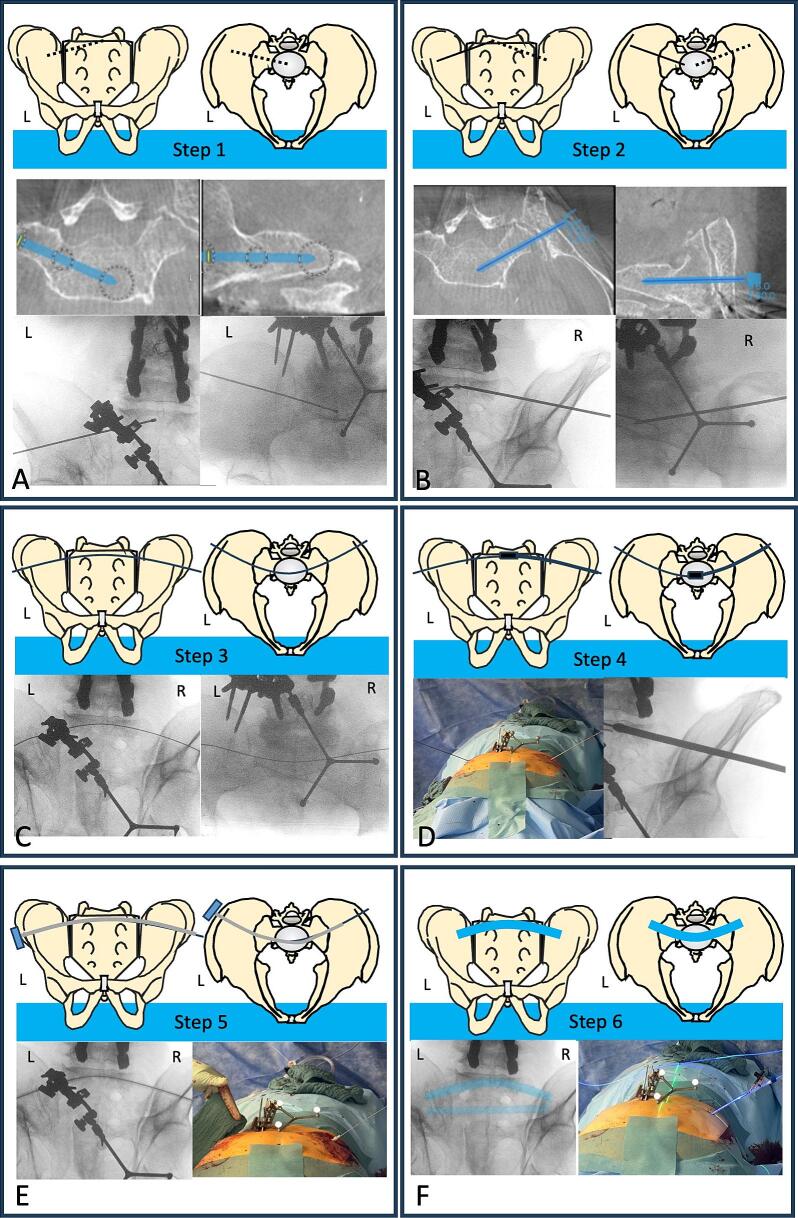
The arc-technique is shown step by step. A: shows the illustration of the guide-wire placement of the left side. The intraoperative 3D-navigation planning is shown which helped to find the correct corridor. A 2.8 × 450 mm guide-wire was placed and drilled using a 5.0 mm cannulated drill. B: shows the illustration of the guide-wire placement of the right side. The intraoperative 3D-navigation planning is shown which helped to find the correct corridor. A 2.8 × 450 mm guide-wire was placed and drilled using a 5.0 mm cannulated drill. C: shows the placement of a 2.0 mm ball-tip guide-wire which crossed from side to the other. D: Shows the reaming over the 2.0 mm ball-tip guide-wire up to the 8.5 mm flexible drill, followed by changing the 2.0 mm to a 1.2 mm ball-tip guide-wire. The clinical image shows both ends of the guide-wire after changing for the 1.2 mm guide-wire. E: Shows the placement of the implant sheath, which is radiologically confirmed and was removed after inserting the implant in it. The clinical image shows. F: Shows the fully filled and cured photodynamic nail (highlighted blue). (For interpretation of the references to colour in this figure legend, the reader is referred to the web version of this article.)
Step 2: a second oblique 2.8 × 450 mm iliosacral guide-wire was also placed with the tip in the right S1 body, to converge at the vertebral midline (Fig. 2 B).
Steps 3 & 4: After drilling with a 5.0 mm drill bit on both sides over the 2.8 mm guide-wire, the guide-wire was replaced by a 2.0 mm ball-tip guide-wire to allow further transiliosacral drilling up to 8.5 mm diameter with a flexible drill bit (IlluminOs, USA) (Fig. 2C, D).
Step 5: Finally, the 2.0 mm guide-wires were removed and a 1.2 mm ball-tip guide wire was advanced transiliosacrally (Fig. 2E). After radiological conformation the implant sheath was inserted.
Step 6: A 9 × 160 mm photodynamic nail was placed into the sheath, filled with fluid-monomer and cured with blue light for 500 s using the light box provided by the manufacturer (IlluminOss, USA) (Fig. 2F).

Step 1: using the 3D navigation guide, an oblique 2.8 × 450 mm iliosacral guide-wire (J&J Medtech, USA) was placed on the left side in the S1 body (Fig. 2A).

### S2 corridor and left superior pubic ramus

**Fig. 3:**
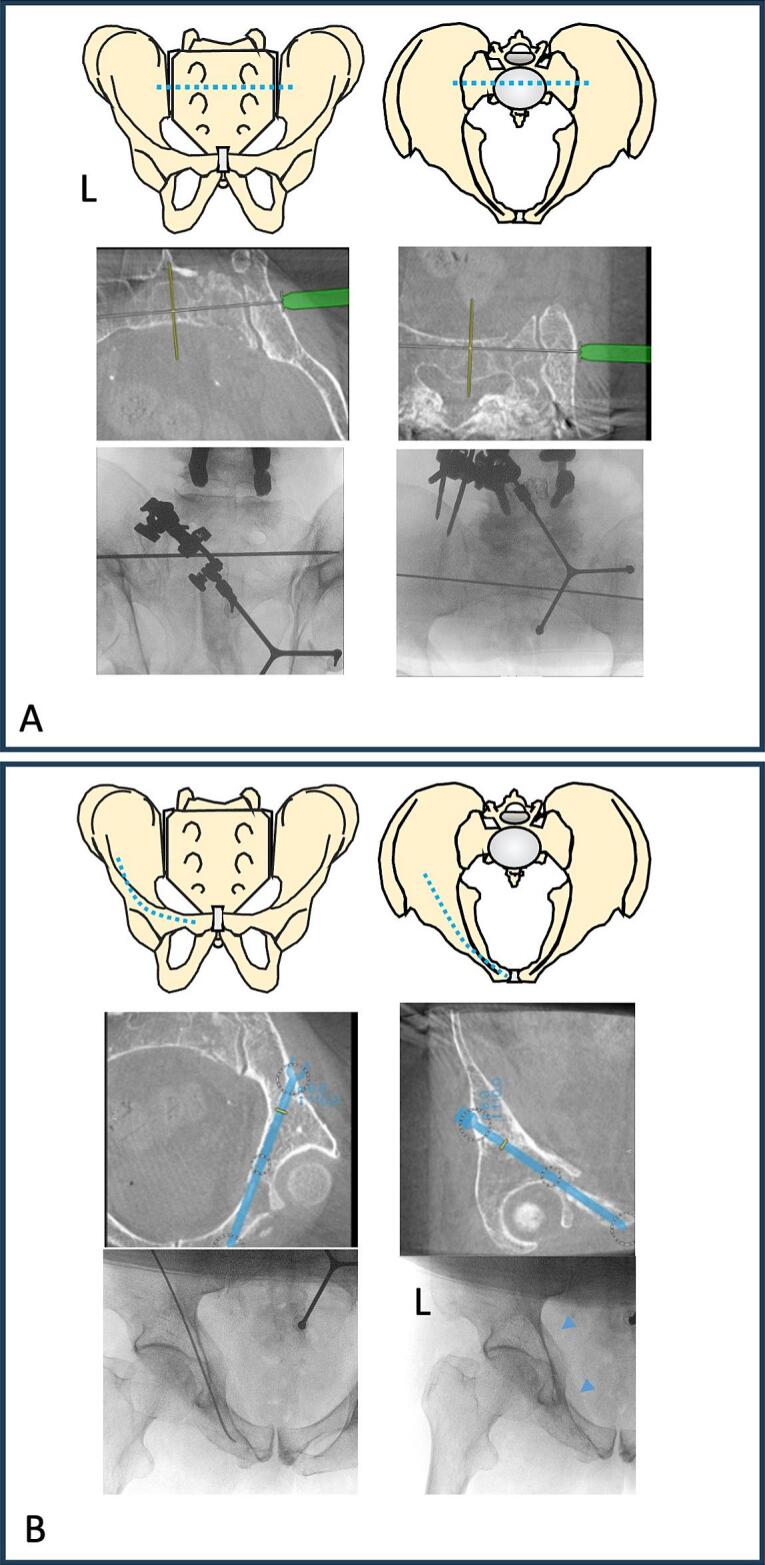
A: shows the placement of the S2 transiliosacral photodynamic nail. Therefore, the corridor was planned using the 3D-navigation, afterwards a 2.8 × 450 mm guide-wire was placed and after drilling finally changed to a 1.2 ball-tip guide-wire for implant placement. B: shows the placement of the superior pubic ramus screw. The intraoperative 3D-navigation planning and the placement of a elastic nail to get into the curved corridor. After drilling the implant sheath was inserted (marked by the blue arrows). (For interpretation of the references to colour in this figure legend, the reader is referred to the web version of this article.)

To address the osteolysis in S2 and the superior pubic ramus photodynamic nails surgical techniques already utilized for percutaneous screw placement were used. Therefore, using a navigation-guided drill-guide to place a 2.8 × 450 mm guide wire in the S2 corridor (Fig. 3A). A 5.0 mm cannulated drill bit was used to drill transiliosacral and a with a depth gauge the length of 160 mm was confirmed. The guide wire was sequentially exchanged—first for a 2.0 mm ball-tip guide-wire facilitating flexible drilling up to 8.5 mm and then changing for a 1.2 mm ball-tip guide-wire, which is mandatory to place a 9 × 160 mm and subsequent the implant sheath was placed. Following sheath positioning, the photodynamic nail (IlluminOss, USA) was inserted, the sheath removed and with light-curable monomer, and polymerized for 500 s at 450 nm using the manufacturer's light box.

The left superior pubic ramus was stabilized next. Therefore a 2.0 mm elastic nail (Synthes, USA) served as a guide-wire for establishing the corridor. The entry point was determined using the 3D-navigation system and in prone position, can usually be found near the S2 entry point, allowing the usage of the same skin incision. After placing the 2.0 mm elastic nail as guide-wire drilling using the 5.0 mm cannulated drill was performed (Fig. 3B). Afterwards the corridor was prepared for the photodynamic nail using the flexible drill up to 8.5 mm and the guide-wire, again was changed to 1.2 mm to allow the placement of the implant sheath. A 9 × 120 mm photodynamic nail was prepared and cured as described for the S1 and S2 corridor.

Finally wounds were irrigated and closed with interrupted sutures.

### Surgical time, blood loss and radiation exposure

The surgical time was 182 min. The time required for light curing the implants was 1500 s. The total area dose product was 3015 cG*cm^2^. The estimated blood loss was 30 ml.

### Postoperative outcome

**Fig. 4:**
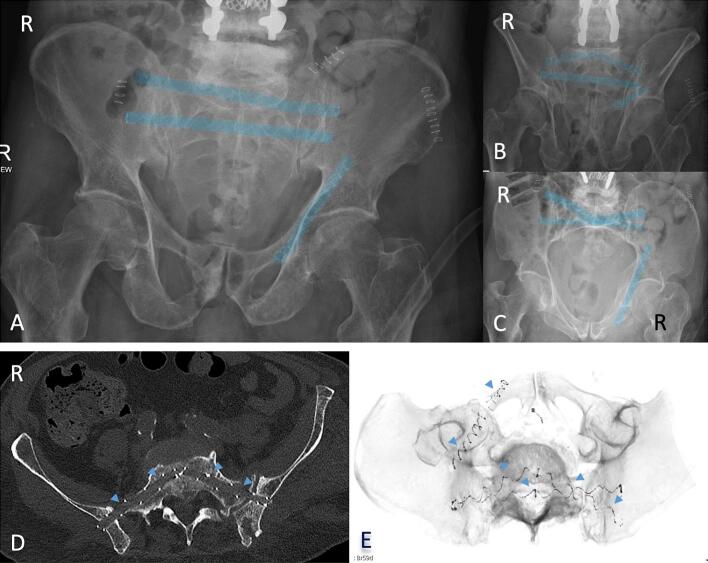
postoperative X-rays of the patient showing the radiopaque spiral of the implant. A: a.p. view of the pelvis, B: outlet-view of the pelvis, C: inlet-view of the pelvis; D: postoperative axial CT-scan, the photodynamic nail is marked by blue arrows; E: 3D-reconstruction of the postoperative CT-scan, all photodynamic implants are marked by blue arrows. (For interpretation of the references to colour in this figure legend, the reader is referred to the web version of this article.)

Postoperative imaging with X-ray (Fig. 4A-C) and CT-scan (Fig. 4D, E) confirmed satisfactory implant placement. The patient began full weight-bearing on day one, with pain reduction from VAS 7 pre-op to VAS 2 by day seven, mirroring results from similar series [15], [16].

## Discussion

Stabilization of fragility fractures involving the pelvic ring remains complex, particularly in patients with sacral dysmorphism—reported in 14–64% of cases [17], [18], [19], [20]. Moreover, 25% of patients may lack a safe S1 transiliac corridor [17]. Anatomical limitations may prevent standard screw placement in S1 in over 25% of cases and also reduced bone mass density can be found in dysmorphic sacra [4], [19], [20], [21], [22]. To increase intraoperative safety, 3D-navigation to enhance screw placement can be used [23]. To increase the biomechanical stability of the osteosynthesis construct, ascending S1 screws or cement augmentation have been used. Furthermore, in vitro studies confirmed that longer screws and triangular constructs improve stability, although cement augmentation offers mixed results [24], [25], [26], [27], [28], [29], [30]. Zakariaee et al., showed that a curved implant design could increase the stability due to possible larger diameter and implant length [31]. Therefore, novel implant options with a curved design like the CurvaFix have been developed to address the challenges of stabilizing posterior pelvic ring injuries, particularly in elderly patients with osteoporotic bone [13], [14]. Unlike traditional straight screws, the CurvaFix implant can follow the natural curvature of pelvic bone corridors, such as the supraacetabular, sacroiliac, and trans-sacral pathways. Early clinical experience suggests enhanced biomechanical stability and promote early mobilization in patients with fragility fractures of the pelvis [13], [14]. However, large-scale randomized trials are still needed to establish the long-term clinical benefits and cost-effectiveness of the CurvaFix system compared to traditional fixation strategies. Additionally, the implant is currently only available in the United States of America. To use the biomechanical benefits of an arc-like implant, this case report shows the first use of a flexible photodynamic nail, in a minimally invasive stabilization technique. Vegt et al., showed proper axial load-sharing properties for photodynamic implants [32]. The arc-technique using a photodynamic nail, as demonstrated in this case, offers a feasible and potentially robust solution.

The time for surgery and radiation dosage required were both increased in this first case. Though, comparable to other fluoroscopic procedures like angiography, with experience of this case surgical time and radiation dosage will decline for this technique. Comparative biomechanical analyses with other techniques are warranted in future research. The arc-technique described here enables stabilization even in dysmorphic and metastatic bone using a construct that bridges the iliac wings via a transiliosacral route [33]. This may offer improved load distribution and implant security according to previous studies [31], [33], however further biomechanical and clinical research is required in the future.

## Conclusions

The arc-configuration with photodynamic implants represents a promising alternative for managing fragility fractures in dysmorphic sacra. Its minimally invasive profile and adaptability to compromised bone offer a feasible strategy, warranting further biomechanical and clinical validation.

## CRediT authorship contribution statement

**Tobias Fritz:** Writing – original draft, Visualization, Project administration, Methodology, Investigation, Data curation, Conceptualization. **Emmanouil Liodakis:** Writing – review & editing, Conceptualization. **Jonas Gras:** Writing – review & editing. **Marcel Orth:** Writing – review & editing, Conceptualization. **Antonius Pizanis:** Supervision, Conceptualization. **Marcus Örgel:** Writing – review & editing. **Tim Pohlemann:** Validation, Supervision, Conceptualization. **David Osche:** Writing – original draft, Investigation, Conceptualization.

## Informed consent statement

Informed consent was obtained from all subjects involved in the study. Written informed consent has been obtained from the patients to publish this paper.

## Institutional review board statement

Ethical review and approval were waived for this study due to description of a surgical technique without a full medical study.

## Funding

No funding was received for this Case Report & Technical Note.

## Declaration of competing interest

PD Dr. Tobias Fritz – Consultancies for IlluminOss Inc. <10,000 €.

Prof. Dr. Emmanouil Liodakis – Associate Editor of Trauma Case Reports.

Prof. Dr. Tim Pohlemann.

Prof. Dr. Marcel Orth – no conflict of interest.

Dr. med. David Osche – no conflict of interest.

Jonas Gras – no conflict of interest.

PD Dr. Marcus Örgel – no conflict of interest.
